# Dosimetric Impact of Interfractional Variations for Post-prostatectomy Radiotherapy to the Prostatic Fossa—Relevance for the Frequency of Position Verification Imaging and Treatment Adaptation

**DOI:** 10.3389/fonc.2019.01191

**Published:** 2019-11-08

**Authors:** Mona Splinter, Tilman Bostel, Ilias Sachpazidis, Tobias Fechter, Constantinos Zamboglou, Oliver Jäkel, Peter E. Huber, Jürgen Debus, Dimos Baltas, Nils H. Nicolay

**Affiliations:** ^1^Medical Physics in Radiation Oncology, German Cancer Research Center, Heidelberg, Germany; ^2^Clinical Cooperation Unit “Radiation Oncology, German Cancer Research Center, Heidelberg, Germany; ^3^Heidelberg Institute of Radiation Oncology (HIRO), National Center for Radiation Research in Oncology (NCRO), Heidelberg, Germany; ^4^Department of Radiation Oncology, Heidelberg University Hospital, Heidelberg, Germany; ^5^Department of Radiation Oncology, University Medical Center Mainz, Mainz, Germany; ^6^Department of Radiation Oncology, Medical Center - University of Freiburg, Freiburg, Germany; ^7^German Cancer Consortium (DKTK), Partner Site Freiburg, German Cancer Research Center, Heidelberg, Germany

**Keywords:** prostate cancer, prostatic fossa, post-prostatectomy radiotherapy, image-guided radiotherapy, dosimetry, organs-at-risk, normal tissue complication probability

## Abstract

**Background and purpose:** To analyze divergences between the planned and applied treatment doses for post-prostatectomy radiotherapy to the prostatic fossa on a voxel-by-voxel basis based on interfractional anatomic variations and imaging frequency.

**Materials and methods:** For 10 patients receiving intensity-modulated postoperative radiotherapy to the prostatic fossa, position verification was carried out by daily in-room CT imaging in treatment position (340 fraction CTs). Applied fraction doses were recalculated on daily CT scans, and treatment doses were accumulated on a voxel-by-voxel basis after deformable image registration. To simulate weekly imaging, derived weekly position correction vectors were used to rigidly register all daily scans of the respective treatment week onto the planning CT before dose accumulation. Detailed dose statistics of the prescribed and applied treatment doses were compared in relation to the frequency of position verification imaging. Derived NTCP and P_injury_ values were calculated for the rectum and bladder.

**Results:** Despite a large variability in the pelvic anatomy, daily CT-based patient repositioning resulted in largely negligible deviations of the analyzed dose-volume, conformity, and uniformity parameters from the planned doses for post-prostatectomy radiotherapy, and only the bladder exhibited significant increases in the accumulated mean and median doses. Derived NTCP for the applied doses to the rectum and bladder and P_injury_ values did not significantly deviate from the treatment plan. In contrast, weekly CT-based repositioning resulted in significant decreases of the PTV coverage and dose conformity as well as large deviations of the applied doses to the rectum and bladder from the planned doses. Consecutively, NTCP for the rectum and P_injury_ were found falsely reduced for weekly patient repositioning.

**Conclusions:** Our data indicate for the first time in a voxel-by-voxel analysis that daily imaging is required for reliable adaptive delivery of intensity-modulated radiotherapy to the prostatic fossa. This work will help guiding adaptive treatment strategies for post-prostatectomy radiotherapy.

## Introduction

Depending on tumor staging and biology, prostatectomy and radiotherapy constitute the two key local treatment modalities for prostate cancer patients, and after surgical tumor removal, additional postoperative irradiation to the prostatic fossa is indicated for patients at high risk of tumor recurrence, e.g., in case of locally advanced cancers, incomplete resection or adverse PSA dynamics (1–5). The advent of modern radiotherapy techniques like image-guided and intensity-modulated radiotherapy (IMRT) has resulted in decreased late treatment-related gastrointestinal and genitourinary toxicities, thus providing a scope for dose escalation (6, 7). However, the use of high-precision radiotherapy modalities for post-prostatectomy treatment of the prostatic fossa strongly depends on the pelvic anatomy with inter- and intra-fractional variations of the bladder and rectum, making the treatment more susceptible to dosimetric inaccuracies (8, 9). While regular image guidance by cone-beam CT (CBCT) is widely available and helps to reduce anatomy-dependent inaccuracies, the low soft tissue contrast of this imaging means often impairs accurate patient repositioning, and additional tools for positional control such as implanted fiducials that are used for the radiotherapy of primary prostate cancers cannot be utilized for postoperative treatment. Therefore, patient repositioning is often carried out according to the pelvic bony anatomy, and anatomic and volumetric alterations of the rectum and bladder often cannot be taken into account. For definitive radiotherapy to the prostate gland, previous work based on weekly CT scans and rigid registration has demonstrated significant aberrations of the applied treatment doses from the dose prescriptions both for IMRT and proton radiotherapy (10, 11). It has been suggested that the postoperative situation makes prostate cancer patients even more susceptible to anatomic and volumetric alterations, and the resulting implications of these interfractional variations for the dose applied to the prostatic fossa and the surrounding organs-at-risk are insufficiently understood (12). No data are available that assessed these variations based on daily high-quality imaging and elastic registration algorithms.

For this analysis, we calculated interfractional variations in postoperative pelvic anatomy and quantified resulting deviations of the applied from the planned treatment doses using daily diagnostic CT scans performed in treatment position immediately before each radiotherapy fraction. Furthermore, the dosimetric impact of the anatomic variability was analyzed in relation to the frequency of position verification imaging. These data will help to guide adaptive re-planning strategies for postoperative radiotherapy to the prostatic fossa.

## Materials and Methods

### Patient Selection

Ten consecutive patients received post-prostatectomy radiotherapy to the prostatic fossa at the German Cancer Research Center and were included in this analysis. All patients presented with a postoperative PSA increase exceeding 0.2 ng/mL according to the guidelines of the American Urological Association as well as a PSA doubling time >6 months and an initial Gleason Score below 8 (13, 14). The analyses are in accordance with the Declaration of Helsinki (Seventh Revision, 2013) and were approved by the Independent Ethics Committee of the Medical Faculty of the University of Heidelberg, Germany (S-380/2017).

### Treatment Planning and Delivery

The clinical target volume (CTV) covered the prostatic fossa as defined by the guidelines of the Radiation Therapy Oncology Group (RTOG) (15). The CTV was expanded by 7 mm to create a planning target volume (PTV), and the prescribed dose was 68 Gy in 34 fractions of 2 Gy. Radiotherapy planning was performed with the RayStation planning system (RaySearch Laboratories, Stockholm, Sweden), and dose constraints to the organs-at-risk (OAR) were defined based on the Quantitative Analyses of Normal Tissue Effects in the Clinic (16–18). Patients were instructed to present to daily treatment with an empty bowel and a comfortably filled bladder, and patient immobilization for treatment was carried out using a ProStep™ pelvic and lower extremity support (Elekta, Stockholm, Sweden). Treatment was applied as step-and-shoot IMRT on an Artiste linear accelerator (Siemens, Erlangen, Germany) using 9 co-planar fields.

### Daily CT Imaging

Patients were immobilized as described above and positioned on the treatment couch based on tattooed skin markers that were applied during the planning CT. The treatment couch was then rotated into an in-room CT scanner (Primatom, Siemens OCS, Malvern, USA) that was part of the linear accelerator setup and located at a 90° angle in the treatment suite. All patients received a daily diagnostic quality CT scan in treatment position as positional verification imaging. After the scan, the couch was re-rotated to the linear accelerator with no manipulation to the patient setup. All scans were taken to the same specifications as used for the individual planning CT examinations for each patient, and the in-room CT scanner was approved for treatment planning scans by the respective regulatory authorities.

### Analysis of Variations and Simulation of Imaging Frequency

Contouring of target volumes and OARs was carried out by a board-certified radiation oncologist on the planning CTs and on all corresponding daily verification scans according to current guidelines (15). Daily position verification imaging was rigidly registered to the planning CT, and resulting individual doses of each treatment fraction were calculated based on the respective daily CT scans, and resulting daily dose distributions were mapped onto the planning scans. Daily doses were accumulated for each voxel using the deformable image registration module in RayStation and compared to the dose distribution of the treatment plan (19). Weekly verification imaging was simulated by deriving position correction vectors from the first CT scan of each treatment week (days 1, 6, 11, 16, 21, 26, and 31) and applying this vector to rigidly register the five daily scans of each treatment week to the planning scan. Daily dose distributions resulting from daily anatomic variability and weekly CT-based repositioning were calculated and mapped onto the planning CT before accumulation for all treatment fractions.

### Dosimetric Analyses

The applied 3D dose distribution based on daily accumulation was compared to the planned using dose-volume indices, including mean dose (D_mean_) and doses as x % volume (D_x_) as well as treatment volume at the x Gy dose level (V_x_). Dose conformity was assessed for the prescribed doses by the conformity (CI) and conformal (COIN) indices, and uniformity was quantified using established parameters EUD and gEUD (see Annex I) (20, 21). Additionally, normal tissue complication probabilities (NTCP) for the relevant OARs and the total probability of normal tissue injury (P_injury_) for rectum and bladder were calculated and compared (see Annexes II, III). The distribution of the applied doses was compared within the region receiving more than 10% of the maximum dose using 3D gamma analyses to the clinical tolerance level of 3%/3 mm (22).

### Statistical Analysis

Dose volume indices of planned and applied doses were compared by Wilcoxon signed-rank test with corresponding two-sided confidence intervals using in-house software developed in Python (https://www.python.org). A *P*-value < 0.05 was considered statistically significant.

## Results

### Anatomic Variability

**Figure 1:**
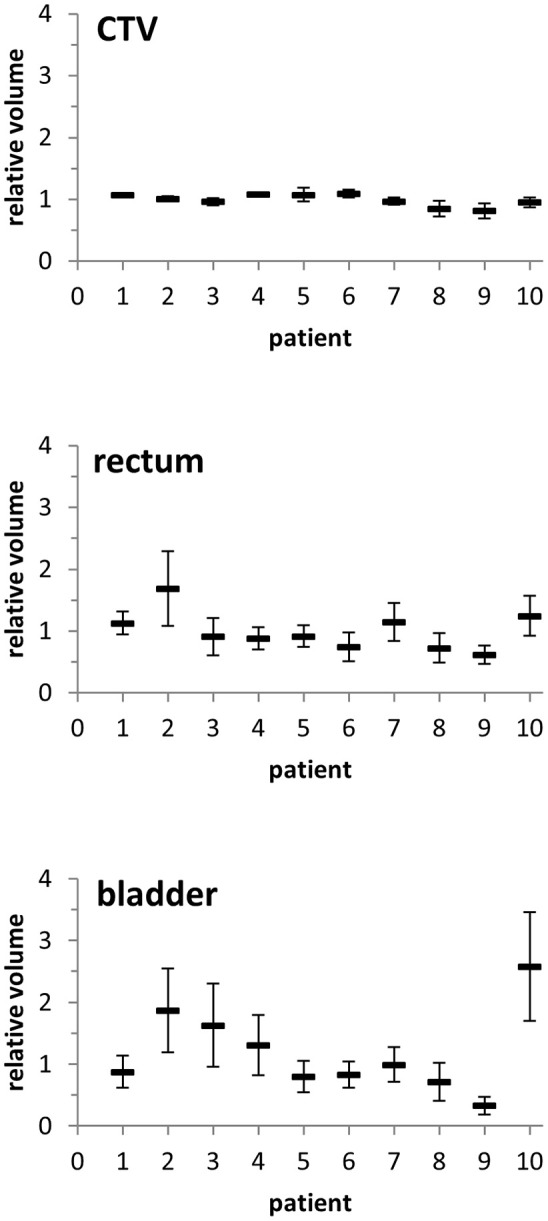
Relative volumes of the CTV, rectum, and bladder of each patient during the course of radiotherapy as compared to the volumetry from the planning CT. Error bars represent standard deviation.

Patients demonstrated notable anatomic and volumetric changes during the course of postoperative radiotherapy. CTVs were very consistent at treatment planning for all analyzed patients and ranged between 78.1 and 90.4 ml. During the course of treatment, the volumes remained very stable with a relative volume difference between the planning CT and the treatment scans of 1.1 ± 9.7% (Figure 1, Supplementary Figure 1). Pre-treatment rectal volumes varied between 36.4 and 112.4 ml and decreased by on average 9.7 ± 32.3% during radiotherapy (Supplementary Figure 1). Expectably, the bladder showed the strongest variability with pre-treatment volumes between 108.5 and 431.2 ml. During radiotherapy, average bladder volumes were found reduced by 19.1 ± 66.6%. For the individual patients, considerable differences in rectum and bladder volumes were observed during radiotherapy.

**Figure 2:**
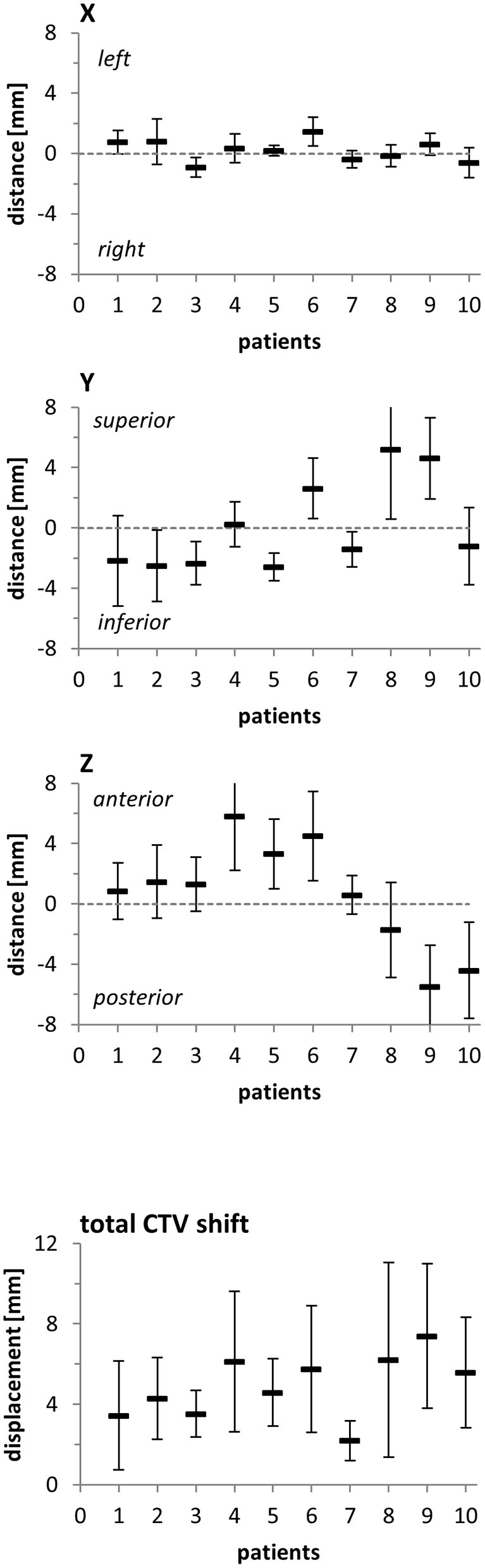
Distance of the CTV's geometric center between the planning CT and the fractional CTs in all three spatial directions and resulting total CTV shift for each patient included in this analysis. Error bars represent standard deviation.

To quantify potential implications of the observed anatomic variations on the CTV, its geometric center was compared between the planning CT and the daily scans following individual rigid registrations based on the surrounding bony structures. The average lateral CTV displacement (X shift) was 0.2 ± 0.2 mm (fractional range: −0.4 to 0.9 mm), and individual patients demonstrated only small effects during their daily treatment fractions (individual range: −0.9 to 1.5 mm) (Figure 2, Supplementary Figure 2). Shifts in the superior-inferior direction (Y shifts) were on average 0.4 ± 0.8 mm (fractional range: −1.3 to 1.5 mm) with considerably larger variability for individual patients (individual range: −2.6 to 5.2 mm). Changes in the anterior-posterior direction (Z shifts) amounted to an average shift of 0.6 ± 0.7 mm (fractional range: −0.7 to 2.1 mm) with strong inter-individual variability (individual range: −5.5 to 5.8 mm). As the PTV margin was set at 7 mm, evaluation of daily verification CTs did not require treatment adaptation or re-planning due to interfractional variability for any analyzed patient.

### Impact of Anatomic Variability on Dose Distribution

**Figure 3:**
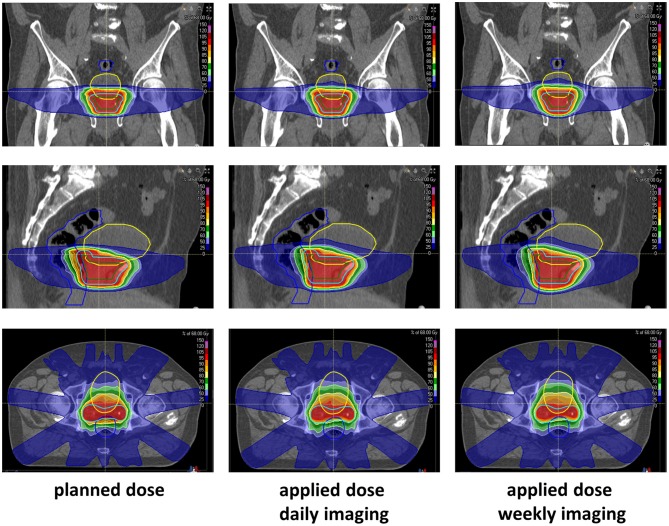
Representative CT imaging demonstrating the planned and accumulated doses based on daily or weekly CT-based repositioning.

**Figure 4:**
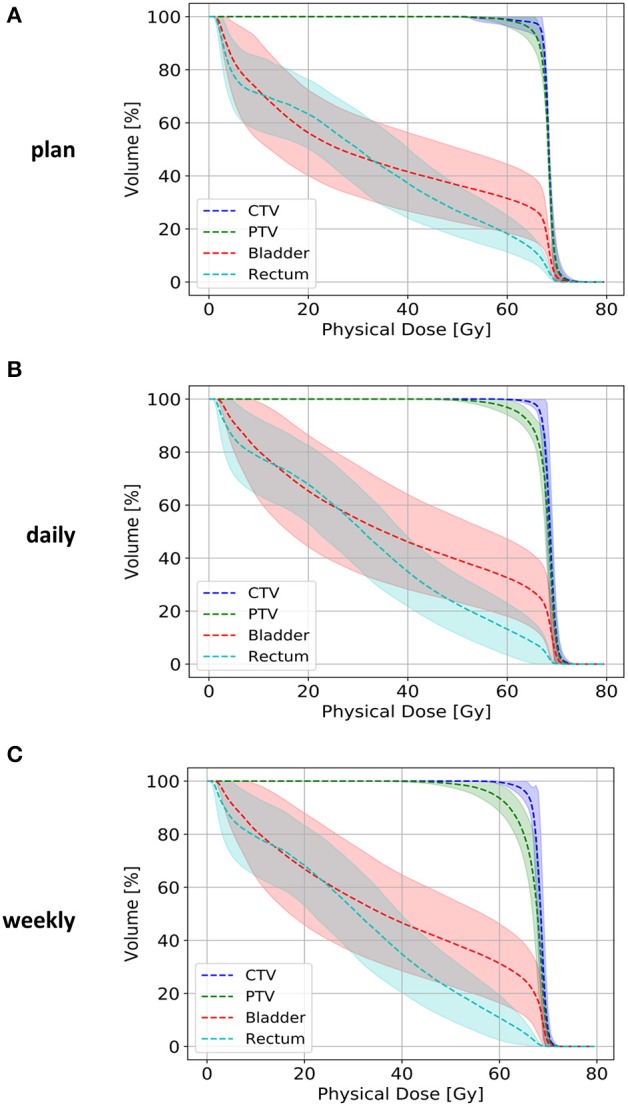
Dose-volume histograms for the CTV (blue line), PTV (green line), rectum (light blue line), and bladder (red line) for the treatment plans **(A)** and accumulated doses after daily **(B)** or weekly **(C)** repositioning. Lighter-colored bands represent the 95% confidence interval of each dose-volume curve.

Planned and applied doses were compared regarding the CTV, PTV, rectum, and bladder (Figure 3). As also demonstrated in the volumetric assessment, the bladder exhibited the largest variability between planned and accumulated doses, and the deviations were most pronounced in the middle dose range (Figure 4). All differences in the dose-volume indices between planned and applied doses are summarized in Table 1. The largest dosimetric deviations in the CTV were observed for the V_68_ with a non-significant average increase of 11% for the accumulated doses (*p* = 0.431). In contrast, the applied D_2_, D_50_, D_mean_, and D_98_ varied <2% from the corresponding parameters in the treatment plan. No significant deviations in the uniform dose parameters EUD and gEUD or the conformity indices CI and COIN were measured between the accumulated and planned doses. In the PTV, the applied D_98_ was found significantly reduced by an average of 7% (*p* = 0.037), while all other accumulated dosimetric parameters only varied non-significantly from those of the treatment plan.

**Table 1:** Average relative deviation (mean values and standard deviation) of the applied from the planned dose-volume indices for daily or weekly CT-based repositioning.

		**Daily imaging**	***P-*value**	**Weekly imaging**	***P-*value**
CTV	D98 (Gy)	0.01 ± 0.06	0.922	0.00 ± 0.08	0.675
	D50 (Gy)	0.01 ± 0.01	0.322	0.00 ± 0.01	0.625
	Dmean (Gy)	0.00 ± 0.01	0.322	0.00 ± 0.01	0.769
	D2 (Gy)	0.00 ± 0.01	0.193	−0.01 ± 0.01	0.037^*^
	V68 (%)	0.11 ± 0.54	0.431	0.02 ± 0.59	0.921
	V64.6 (%)	0.01 ± 0.04	0.932	−0.01 ± 0.06	0.441
	EUD (Gy)	0.01 ± 0.02	0.322	0.00 ± 0.03	0.769
	gEUD (Gy)	0.02 ± 0.04	0.492	0.01 ± 0.05	0.695
	CI	0.01 ± 0.49	0.625	−0.24 ± 0.49	0.375
	COIN	0.23 ± 0.60	0.275	0.37 ± 0.72	0.193
PTV	D98 (Gy)	−0.07 ± 0.09	0.037^*^	−0.11 ± 0.12	0.014^*^
	D50 (Gy)	0.00 ±0.01	0.375	−0.01 ± 0.01	0.275
	Dmean (Gy)	−0.01 ± 0.01	0.275	−0.02 ± 0.02	0.037^*^
	D2 (Gy)	−0.01 ± 0.01	0.160	−0.01 ± 0.01	0.006^*^
	V68 (%)	0.03 ± 0.49	0.625	−0.20 ± 0.51	0.375
	V64.6 (%)	−0.05 ± 0.08	0.084	−0.15 ± 0.13	0.009^*^
	EUD (Gy)	−0.02 ± 0.03	0.160	−0.05 ± 0.06	0.027^*^
	gEUD (Gy)	−0.04 ± 0.08	0.193	−0.09 ± 0.13	0.049^*^
	CI	0.05 ± 0.45	0.625	−0.20 ± 0.44	0.375
	COIN	[n] 0.53	0.625	−0.21 ± 0.56	0.492
Rectum	D50 (Gy)	0.03 ± 0.19	0.557	0.02 ± 0.22	0.557
	Dmean (Gy)	0.01 ± 0.14	0.845	0.00 ± 0.14	1.000
	D2 (Gy)	−0.02 ± 0.02	0.019^*^	−0.04 ± 0.03	0.002^*^
	V70 (%)	−0.52 ± 1.94	0.204	−0.98 ± 1.34	0.022^*^
	V50 (%)	−0.16 ± 0.28	0.160	−0.20 ± 0.30	0.049^*^
	V40 (%)	−0.07 ± 0.19	0.375	−0.07 ± 0.22	0.557
	EUD (Gy)	−0.05 ± 0.07	0.105	−0.08 ± 0.07	0.002^*^
	gEUD (Gy)	−0.06 ± 0.08	0.049^*^	−0.10 ± 0.08	0.002^*^
Bladder	D50 (Gy)	0.18 ± 0.23	0.037^*^	0.22 ± 0.28	0.049^*^
	Dmean (Gy)	0.10 ± 0.12	0.019^*^	0.11 ± 0.14	0.037^*^
	D2 (Gy)	0.00 ± 0.01	0.322	−0.01 ± 0.02	0.105
	V70 (%)	0.19 ± 0.92	0.635	−0.50 ± 1.03	0.236
	V55 (%)	0.06 ± 0.12	0.193	0.04 ± 0.16	0.322
	V45 (%)	0.09 ± 0.13	0.064	0.10 ± 0.16	0.064
	EUD (Gy)	0.00 ± 0.03	0.625	−0.01 ± 0.05	1.000
	gEUD (Gy)	0.00 ± 0.04	0.845	−0.02 ± 0.06	0.846

Negative values represent decreases in accumulated doses. CTV, clinical target volume; EUD, equivalent uniform dose; gEUD, generalized equivalent uniform dose; CI, conformity index; COIN, conformal index. Significant P values < 0.05 (^*^) are printed in bold.

The applied D_mean_ to the rectum was reduced by an average of 0.25 Gy compared to the plan, and only the D_2_ deviated significantly from the planned dose with a reduction of 1.51 ± 1.69 Gy (*p* = 0.019). Due to the observed alterations in the bladder volume during postoperative radiotherapy, the applied D_mean_ and D_50_ were found increased by 3.56 ± 4.02 and 5.14 ± 6.51 Gy, respectively (*p* = 0.037 for ΔD_mean_, *p* = 0.019 for ΔD_50_). All other dosimetric parameters for the accumulated bladder dose only deviated non-significantly from the planned dose, and the EUDs for both the rectum and bladder were also found comparable.

The median NTCP values derived from the accumulated doses to the rectum and bladder were 0.87 % (min 0.36%, max 5.88%) and 5.34% (min 0.92%, max 8.98%), respectively, and for the treatment plan were 2.04% (min 0.92%, max 4.52) and 4.99% (min 1.99%, max 7.03%), respectively.

**Figure 5:**
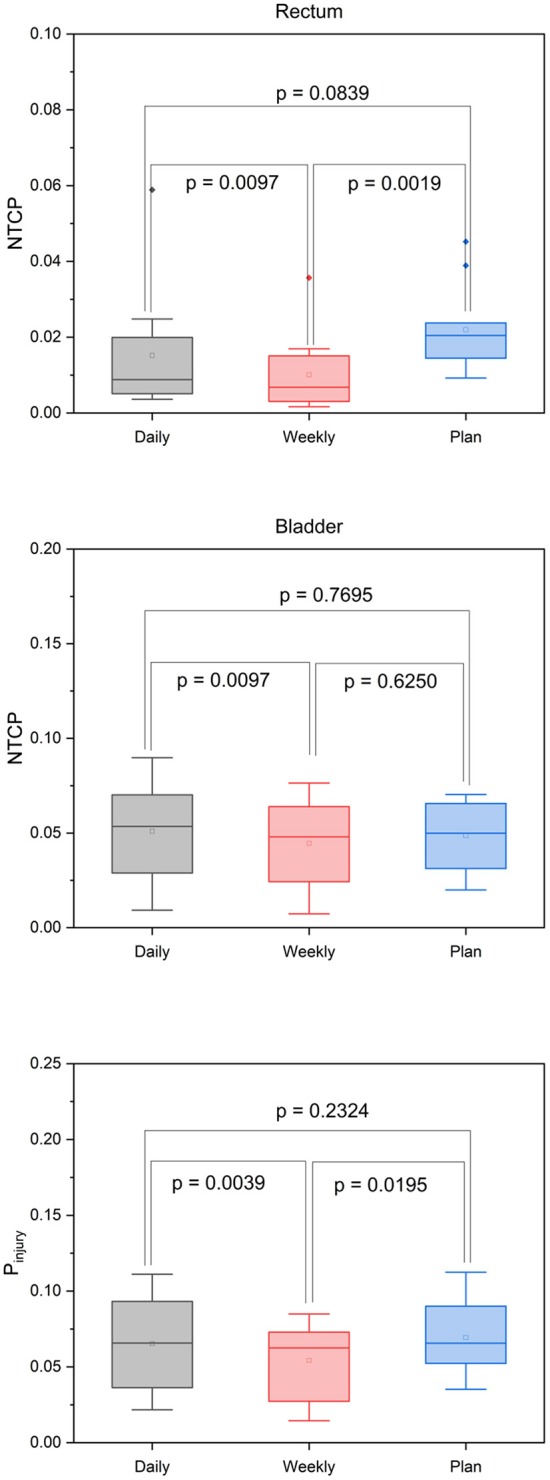
Box-plot diagrams for the NTCP of the rectum and bladder and the P_injury_ values derived from the planned and accumulated doses after daily and weekly CT-based repositioning.

The values resulting from the daily dose accumulation did not significantly differ from the NTCP values calculated from the treatment plans (*p* = 0.084 for the rectum, *p* = 0.770 for the bladder; Figure 5). Similarly, P_injury_ as a means of quantifying overall treatment-related toxicities did not significantly differ between the planned and applied doses (*p* = 0.232).

### Dosimetric Consequences of Daily vs. Weekly Position Verification Imaging

**Figure 6:**
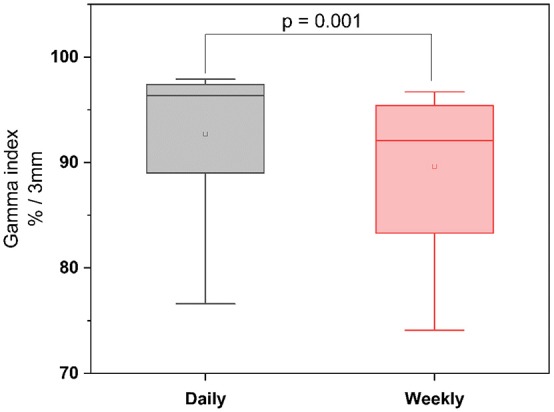
Gamma passing rates after daily and weekly CT-based repositioning to the clinical tolerance level of 3%/3 mm.

To quantify the dosimetric consequences of daily vs. weekly position verification imaging, verification CTs of each treatment week were registered to the planning CT based on the repositioning vector derived from the first weekly verification scan (fractions 1, 6, 11, 16, 21, 26, and 31). Applied daily doses were accumulated and compared to the planned doses and accumulated doses from daily CT-based repositioning. The strongest deviations from the planned dose-volume indices were found in the high dose range with the D_2_ significantly reduced for the CTV (−0.78 ± 0.90 Gy; *p* = 0.037), the PTV (−0.93 ± 0.84 Gy; *p* = 0.006), and the rectum (−2.88 ± 2.40 Gy; *p* = 0.002; Figure 4, Table 1) (Supplementary Table 1). While weekly imaging-based repositioning resulted in only non-significant deviations of the applied CTV doses, several dosimetric indices of the PTV were found significantly reduced upon weekly repositioning, including the D_mean_ (−1.40 ± 1.46 Gy; *p* = 0.037), the D_98_ (−7.13 ± 7.64 Gy; *p* = 0.014), the V64.6 (14.3 ± 12.0%; *p* = 0.009), and the uniformity indices EUD (−3.31 ± 4.35 Gy; *p* = 0.027) and gEUD (−6.09 ± 8.68 Gy; *p* = 0.049). Rectal volumes exposed to 70 and 50 Gy were found significantly lower after weekly repositioning (V_70_: *p* = 0.022; V_50_: *p* = 0.049), and the NTCP values for the rectum were found significantly reduced for the simulated weekly repositioning algorithm compared to both the planned NTCP (*p* = 0.002) and the NTCP derived from daily repositioning (*p* = 0.010; Figure 5). For the bladder, the D_50_ and D_mean_ were found increased by 6.31 ± 7.98 Gy (*p* = 0.049) and 3.67 ± 4.90 Gy (*p* = 0.037) following weekly position verification imaging, respectively. The NTCP for the bladder did not significantly deviate after weekly repositioning in comparison to the planned NTCP or the NTCP derived from daily CT-based positional adaption. The P_injury_ values as calculated from weekly repositioning significantly deviated from both the planned values (*p* = 0.020) or the values derived from daily repositioning (*p* = 0.004). The gamma passing rate to the clinical tolerance level of 3%/3 mm was 3.1% lower for the weekly position verification imaging than that for daily imaging (*p* = 0.001; Figure 6).

## Discussion

Intra- and inter-fractional variations in the pelvic anatomy have long been hypothesized to influence prostate cancer radiotherapy, and the resulting dosimetric impact of these changes has been widely studied. So far, definitive studies have been strongly impaired by the quality and frequency of available positional imaging means and the lack of adequate image registration tools. To the best of our knowledge, this is the first dataset providing a CT-based voxel-wise dosimetric analysis resulting from the interfractional alterations in postoperative intensity-modulated radiotherapy to the prostatic fossa; daily planning-quality in-room CT imaging was utilized prior to each radiotherapy fraction in each patient, and an elastic registration algorithm allowed voxel-by-voxel tracking of the fractional treatment doses.

Our data showed that despite significant interfractional variations in rectal and bladder volume, daily CT-based repositioning led to only minor deviations in the dosimetric parameters concerning the CTV, the PTV and the rectum, while only the mean and median doses to the bladder were significantly increased compared to the treatment plan. In contrast, repositioning based on only weekly verification scans resulted in significant discrepancies between the planned and applied doses to the PTV as well as a significantly lesser dose conformity to the target volume.

Previous datasets analyzing dosimetric implications of variations in the pelvic anatomy for prostate radiotherapy have been mostly based on cone-beam CT (CBCT); however, the low quality of these images has strongly hampered viable dosimetric analyses, and various improvement strategies have been proposed to overcome the clear limitations of CBCT, including enhanced CBCTs utilizing additional filters, portal dose measurements or reliance on rigid registration with apparent limitations for the dose accumulation strategies (8, 23–25). Diagnostic CT to perform voxel-wise dose tracking has so far only been used in the context of proton radiotherapy, and two previous reports analyzing in-room CT data have reported minor deviations in the PTV dose coverage for definitive radiotherapy; the observed dose reductions were mainly attributed to alterations in the rectal volume (26, 27). For definitive IMRT to the prostate gland, only one publication reported dose comparison data derived from daily CT scans and an elastic registration algorithm (28). Despite the considerably stronger impact of anatomic variations after prostatectomy, no data are available for the quantification of dosimetric deviations in postoperative radiotherapy of the prostatic fossa. One previous report analyzed randomly chosen position verification CTs for 10 patients undergoing postoperative radiotherapy and reported significant anatomic variability with the Dice coefficients for the CTV, rectum and bladder ranging at 86.6, 77.3, and 75.4%, respectively (29). However, this study did not perform an accumulation of the fractional doses based on daily imaging, but interpolated the applied doses by using about 10 randomly chosen scans per patient; therefore, definitive conclusions regarding the dosimetric implications of the observed variability in the pelvic anatomy are limited.

It is conceivable that the variable pelvic anatomy and the changes in rectal and bladder filling determine the fractional dose to the prostatic fossa and the adjacent OARs, and interfractional motion of the prostatic bed has been reported in several analyses. A study tracking postoperative clips at the superior and inferior border of the prostatic fossa reported movement of the CTV up to 5 mm in all spatial directions and suggested geographical miss in a significant percentage of patients for isotropic PTV margins or non-daily imaging (30). Two older analyses of small patient cohorts receiving daily megavoltage CT (MVCT) or CBCT suggested that upon daily imaging, the critical anatomic variations of the prostatic fossa remain relatively small for the majority of treatment fractions if repositioning is performed (31, 32). Therefore, the optimal frequency of position verification imaging for a precise and accurate dose delivery has been strongly debated both for definitive and postoperative prostate radiotherapy concepts. While no data are available on this topic for the treatment of the prostatic fossa, a previous study using CBCTs of 20 patients undergoing definitive prostate radiotherapy suggested an improved coverage of the CTV and reduced doses to the rectum by daily positional imaging, although the data quality was limited by the low imaging contrast and the lack of a deformable registration algorithm (33). Similar analyses have suggested that daily imaging may result in better target coverage, allowing a reduction of the PTV margins for daily imaging (34). While these suggestions are mainly derived from definitive treatment concepts, the data presented here back up this suggestion also for postoperative radiotherapy: In our dataset, daily CT-guided repositioning resulted in no significant deviations of the applied fractional doses as compared to the treatment plan for both the CTV and the PTV and led to a better PTV coverage and dose homogeneity than weekly repositioning. Additionally, most dosimetric indices for the rectum and bladder were highly consistent with the treatment plan; however, the clinical significance of these dosimetric improvements needs to be correlated with potential improvements in the relevant clinical outcome parameters. For definitive prostate radiotherapy, two prospective clinical trials have elucidated the patient benefit of daily positional imaging. In a recent French trial, daily CBCTs improved patients' progression-free survival and late rectal toxicity, but correlated with a reduced overall survival (35). A second trial failed to demonstrate any patient impact of a PTV margin reduction concept based on daily CBCT imaging (36).

Current technical developments may further aid the decrease of dosimetric deviations between the applied and the planned treatment doses. For definitive radiotherapy of the prostate, the implantation of radiopaque or electromagnetic fiducial markers is often used to substitute daily CT imaging, and the utilization of electromagnetic motion trackers has also been proposed for post-prostatectomy radiotherapy (37). MR-guided radiotherapy enables real-time tracking of pelvic organs and may hence contribute to minimizing inter- and intrafractional dosimetric deviations (38–40). Also, mathematical concepts have been proposed that guide daily patient positioning based on the already accumulated doses for each treatment day (41). Based on superior imaging modalities like in-room CT or in-room MRI, adaptive treatment strategies may help to provide a daily treatment plan based on the current anatomy. In our dataset, despite daily diagnostic quality CT imaging, the actual mean and median doses to the bladder were significantly higher than planned, and although the clinical implications of this dose increase remain unclear, adaptive postoperative radiotherapy strategies may help to reduce doses to the OARs. This is in contrast with previous data on definitive prostate radiotherapy, where daily imaging resulted in only negligible dosimetric deviations from the treatment plan (28). Voxelwise dosimetric analyses may also help to re-evaluate and potentially revise commonly used PTV margin concepts that have been mainly based on observed interfractional shifts of anatomic landmarks or probabilistic analyses (42).

While our dataset comprises daily diagnostic quality imaging and comprehensive dosimetric information based on a state-of-the-art elastic registration algorithm, our analysis has several limitations. The patient cohort used for this study was relatively small due to the complex logistics associated with the imaging workflow, and the small cohort size may have an impact on the statistical power of our analyses. No intrafractional imaging could be performed in this dataset, and no information was available on anatomic variations during treatment. As the data were generated from clinical procedures, all treated patients were coached about the required rectal and bladder protocol and received regular feedback about their anatomic deviations as assessed by daily CT. This may explain the moderate effects of the interfractional variability on the dosimetric parameters of the rectum and bladder, and routine patients, especially without coaching or daily 3D imaging, may in reality experience significantly higher dose deviations to the OARs. The imaging schedule was simulated by analyzing the first CT scan of every treatment week as routinely done in clinical reality. However, it needs to be noted that this selection process may not adequately represent other non-daily imaging schedules.

Nevertheless, our dataset provides for the first time an in-depth voxel-by-voxel analysis of the dosimetric impact of interfractional variations in post-prostatectomy radiotherapy. These data generated on the basis of daily diagnostic quality positional CT scans and elastic registration-based dose mapping will help to guide imaging frequency and adaptive treatment strategies for postoperative radiotherapy to the prostatic fossa.

## Conclusion

Irrespective of a large variability in the pelvic anatomy, regular rigid patient repositioning based on daily in-room CT imaging resulted in largely negligible aberrations of the applied treatment doses from the planned doses for post-prostatectomy radiotherapy to the prostatic fossa, and only the bladder exhibited increases in the accumulated mean and median doses. However, patient repositioning according to a weekly imaging schedule led to significant decreases in the PTV coverage and dose conformity as well as deviations of the applied doses to the rectum and bladder as compared to the treatment plan. Our data indicate for the first time in a voxel-by-voxel analysis that daily imaging is required for a reliable adaptive delivery of intensity-modulated radiotherapy to the prostatic fossa. This work will help to guide adaptive treatment strategies for post-prostatectomy radiotherapy.

## Data Availability Statement

The datasets generated for this study will not be made publicly available. These are patient data that are protected by German Law and cannot be made available to any person other than the treating physician without expressed written consent by each patient included in this study.

## Ethics Statement

The studies involving human participants were reviewed and approved by Independent Ethics Committee of the Medical Faculty of the University of Heidelberg. The patients/participants provided their written informed consent to participate in this study.

## Author Contributions

TB and NN planned and carried out the treatment. MS, TB, IS, TF, CZ, OJ, DB, and NN analyzed the data. NN wrote the manuscript. IS and DB helped with writing the manuscript. PH and JD helped with data discussion.

### Conflict of Interest

The authors declare that the research was conducted in the absence of any commercial or financial relationships that could be construed as a potential conflict of interest.

## Supplementary Material

Supplementary Figure 1 Mean relative volumes of the CTV, rectum, and bladder for each treatment fraction as compared to the volumetry from the planning CT. Error bars represent standard deviation.

Supplementary Figure 2 Distance of the CTV's geometric center between the planning CT and the fractional CTs in all three spatial directions and resulting total CTV shift for each treatment fraction. Error bars represent standard deviation.

Supplementary Table 1 Median values and interquartile ranges of the dose-volume indices for the treatment plan as well as the accumulated doses based on daily or weekly positional CT imaging.
