# Supplementary material for: Dosimetric Impact of Interfractional Variations for Post-prostatectomy Radiotherapy to the Prostatic Fossa—Relevance for the Frequency of Position Verification Imaging and Treatment Adaptation
Source: Front Oncol. 2019 Nov 8;9:1191. doi: 10.3389/fonc.2019.01191 (PMC6856079; doi:10.3389/fonc.2019.01191)
Supplement: Supplementary file 1 [file Data_Sheet_1.docx]

**Annexes**

***Annex I***

### *The generalized equivalent uniform dose gEUD*

The generalized equivalent uniform dose (*gEUD*) is given for any VOI type (targets and OARs) by the following expression[44]:

$$gEUD=\left( \frac{1}{N}\sum_{i=1}^{N} D_{i}^{\alpha} \right)^{\frac{1}{\alpha}}$$

 (A1)

*N* is the number of dose sample points in the VOI and *D_i_* is the dose at the *i*^th^ calculation point. Generally, *gEUD* is calculated based on the differential DVH of the corresponding VOI and in such a case *N* is the total number of DVH bins.

*gEUD* is based on the physical volumetric dose distribution. To account for the effects of different fractionation at different sampling points within the VOI, the *gEUD_2Gy_* quantity is also used. *gEUD_2Gy_*uses the 2Gy per fraction equieffective volumetric dose distribution as given below:

$${gEUD}_{2Gy}=\left( \frac{1}{N}\sum_{i=1}^{N} {EQD2}_{i}^{\alpha} \right)^{\frac{1}{\alpha}}$$

 (A2)

*EQD2_i_* is the equieffective dose at 2Gy per fraction of a total dose *D_i_* delivered at *d_i_* dose per fraction as this is calculated by:

$${EQD2}_{i}= \frac{D_{i}(1+ \frac{d_{i}}{a/\beta})}{1+ \frac{2 Gy}{a/\beta}}$$

 (A3)

The parameter *α* for a normal tissue(*α>*0)-specific parameter that describes the dose-volume effect of the anatomic structure of interest.

***Annex II***

The response probability *NTCP* for an OAR of volume *V* irradiated with a dose distribution *{D}* is calculated using the relative seriality model [45, 46]**:**

| $NTCP=P\left( \left\{ D \right\},V \right)=\left[ 1-\prod_{i=1}^{M} {(1-{P(D_{i})}^{s})}^{\frac{V_{i}}{V}} \right]^{\frac{1}{s}}$ | **(A5)** |
| --- | --- |

with *P(D_i_)* as the response probability for the OAR having the reference volume and being irradiated to dose *D_i_*:

| $P(D_{i})=e^{-(\frac{{EQD2}_{i}}{D50})(e\gamma-lnln2)}$ | (A6) |
| --- | --- |

where *D_50_* is the equieffective dose *EQD2* resulting to 50% complication probability and *γ* is the maximum value of the normalized dose-response gradient.

*V_i_/V* is the volume fraction being irradiated to dose *D_i_,* and *s* is the parameter which expresses the degree of seriality. Values of *s* close to zero indicate nearly parallel structured organs, where increasing *s* values characterize increasing seriality.

The overall probability of injury *P_I_* is given by:

| $P_{I}=1- \prod_{i=1}^{N_{OAR}} (1-{NTCP}_{i})$ | (A7) |
| --- | --- |

with *NTCP_i_* being the complication probability for the *i*^th^ OAR and *N_OAR_*is the total number of OARs under consideration, here *N_OAR_* = 2 (bladder and rectum). *NTCPs* are calculated from the corresponding DVHs.

***Annex III***

Parameter values for the NTCP (relative seriality model) and gEUD-based calculations are depicted in table A1. The column *s* denotes the relative seriality index. *D_50_* is defined as equieffective dose value *EQD2*.

| **VOI** | ***D_50_***  **(Gy)** | ***γ*** | ***s*** | ***α/β* (Gy)** | **clinical endpoint** | ***α***  **volume effect**  **for *gEUD*[47]** |
| --- | --- | --- | --- | --- | --- | --- |
| Bladder | 80.0 | 2.59 | 1.30 | 3.0 | Symptomatic bladder contracture and volume loss [48, 49] | 6.0 |
| Rectum | 80.0 | 1.79 | 0.75 | 3.0 | Symptomatic proctitis, necrosis, fistula, stenosis [48, 49] | 6.0 |
