# Supplementary figures and images for: Dosimetric Impact of Interfractional Variations for Post-prostatectomy Radiotherapy to the Prostatic Fossa—Relevance for the Frequency of Position Verification Imaging and Treatment Adaptation
Source: Front Oncol. 2019 Nov 8;9:1191. doi: 10.3389/fonc.2019.01191 (PMC6856079; doi:10.3389/fonc.2019.01191)

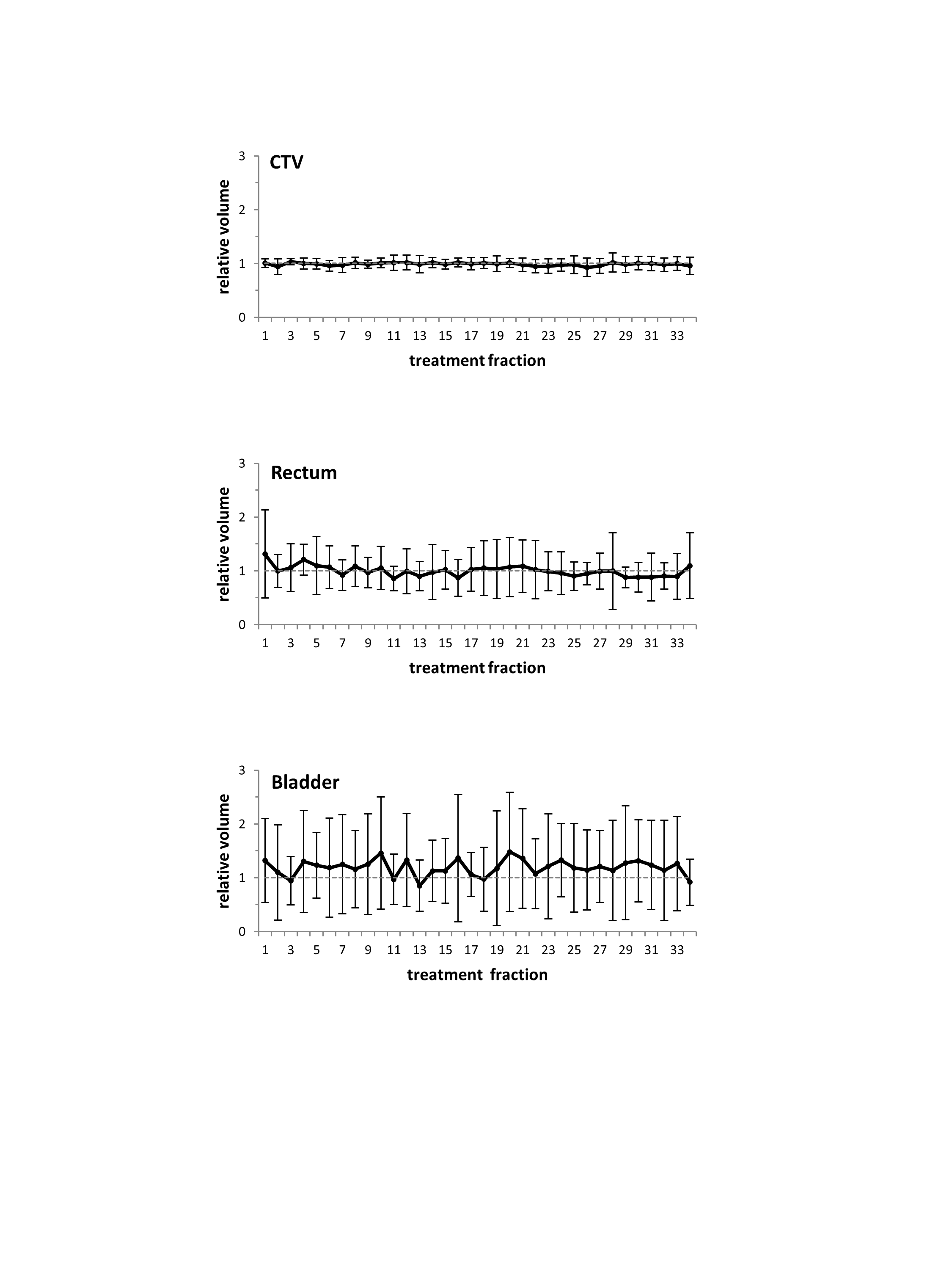

Supplement: Supplementary Figure 1 — Mean relative volumes of the CTV, rectum, and bladder for each treatment fraction as compared to the volumetry from the planning CT. Error bars represent standard deviation. [file Image_1.tif]

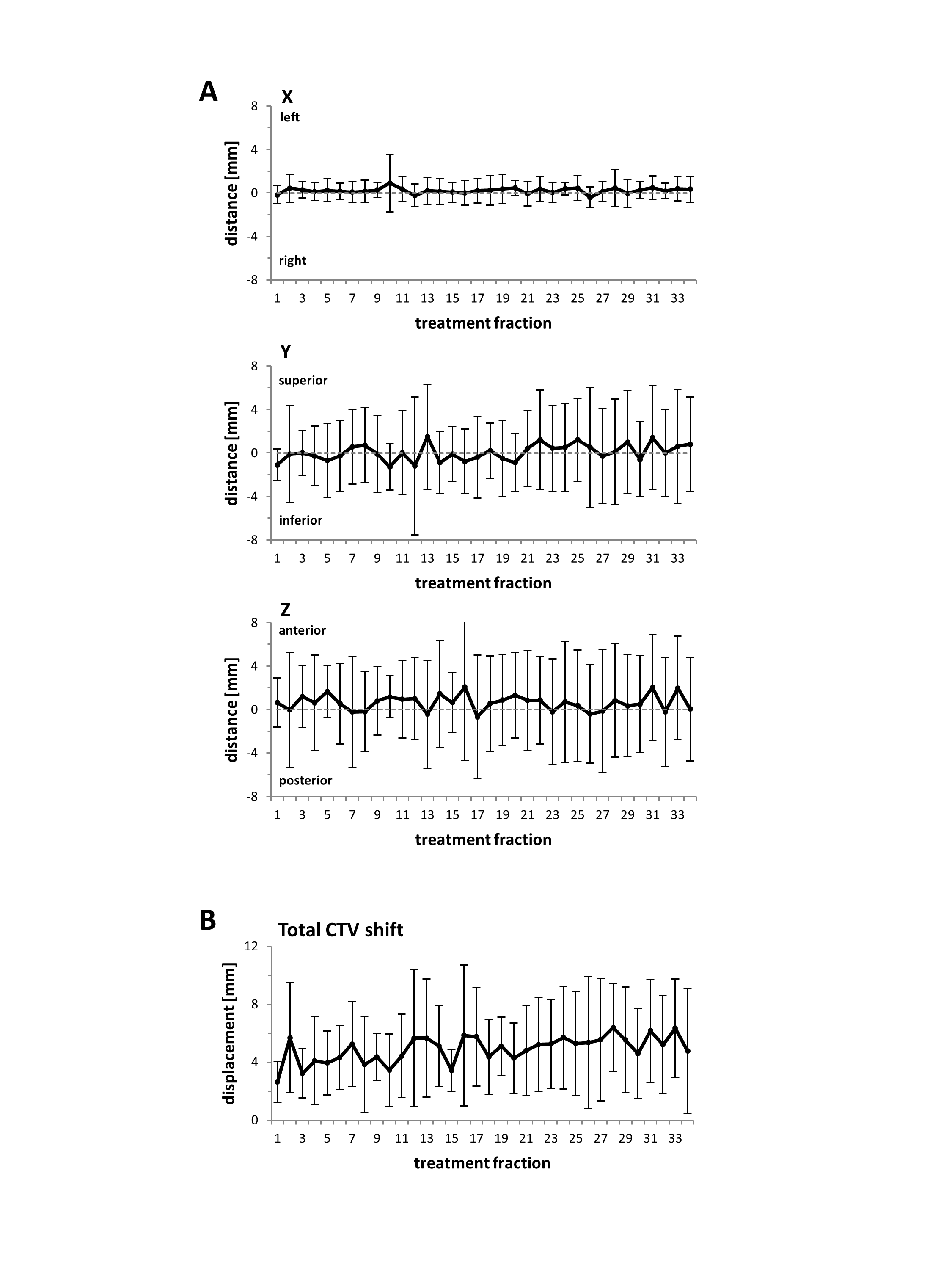

Supplement: Supplementary Figure 2 — Distance of the CTV's geometric center between the planning CT and the fractional CTs in all three spatial directions and resulting total CTV shift for each treatment fraction. Error bars represent standard deviation. [file Image_2.tif]
