# Supplementary material for: Dosimetric Impact of Interfractional Variations for Post-prostatectomy Radiotherapy to the Prostatic Fossa—Relevance for the Frequency of Position Verification Imaging and Treatment Adaptation
Source: Front Oncol. 2019 Nov 8;9:1191. doi: 10.3389/fonc.2019.01191 (PMC6856079; doi:10.3389/fonc.2019.01191)
Supplement: Supplementary Table 1 — Median values and interquartile ranges of the dose-volume indices for the treatment plan as well as the accumulated doses based on daily or weekly positional CT imaging. [file Table_1.DOCX]

|  |  | treatment plan | daily imaging | weekly imaging |
| --- | --- | --- | --- | --- |
| CTV | D98 (Gy) | 66.59 (66.19-66.81) | 65.89 (64.03-66.68) | 64.40 (63.12-65.93) |
|  | D50 (Gy) | 68.08 (68.07-68.23) | 68.44 (67.99-68.87) | 68.41 (67.66-68.69) |
|  | Dmean (Gy) | 68.09 (68.07-68.18) | 68.46 (67.95-68.80) | 68.10 (67.42-68.47) |
|  | D2 (Gy) | 69.85 (69.60-70.82) | 69.95 (69.50-70.38) | 69.71 (68.95-70.09) |
|  | V68 (%) | 56.36 (53.65-61.80) | 78.64 (50.09-83.11) | 70.72 (30.91-81.07) |
|  | V64.6 (%) | 100 (100-100) | 99.83 (97.21-99.97) | 98.05 (94.76-99.86) |
|  | EUD (Gy) | 68.04 (38.01-68.09) | 68.37 (67.86-68.57) | 67.78 (66.99-68.39) |
|  | gEUD (Gy) | 68.03 (67.98-68.10) | 68.40 (67.68-68.61) | 67.52 (66.33-68.57) |
|  | CI | 1.51 (1.31-1.66) | 1.68 (1.28-1.94) | 1.47 (0.55-1.57) |
|  | COIN | 0.22 (0.20-0.25) | 0.30 (0.18-0.39) | 0.33 (0.19-0.46) |
|  |  |  |  |  |
| PTV | D98 (Gy) | 64.06 (63.21-64.95) | 59.85 (57.40-61.40) | 57.46 (52.04-58.88) |
|  | D50 (Gy) | 68.00 (68.00-68.01) | 68.27 (67.87-68.61) | 67.80 (66.88-68.24) |
|  | Dmean (Gy) | 67.83 (67.76-67.89) | 67.50 (66.84-67.89) | 66.16 (65.23-67.59) |
|  | D2 (Gy) | 70.06 (67.71-70.83) | 69.91 (69.39-70.52) | 69.59 (68.67-70.04) |
|  | V68 (%) | 50.29 (50.12-50.45) | 59.74 (43.81-68.38) | 45.21 (17.30-61.37) |
|  | V64.6 (%) | 97.64 (96.01-98.51) | 91.03 (86.92-93.42) | 77.28 (72.89-88.84) |
|  | EUD (Gy) | 67.67 (66.94-67.77) | 66.56 (65.75-67.02) | 65.16 (62.22-66.29) |
|  | gEUD (Gy) | 67.38 (64.50-67.56) | 64.55 (62.56-65.87) | 62.76 (57.05-64.29) |
|  | CI | 0.52 (0.51-0.70) | 0.72 (0.47-0.78) | 0.63 (0.19-0.68) |
|  | COIN | 0.49 (0.37-0.49) | 0.43 (0.35-0.63) | 0.30 (0.14-0.61) |
|  |  |  |  |  |
| Rectum | D50 (Gy) | 25.64 (23.17-34.47) | 26.85 (21.84-35.54) | 26.57 (21.72-36.81) |
|  | Dmean (Gy) | 28.46 (27.62-35.31) | 29.27 (26.80-35.65) | 28.44 (26.11-36.41) |
|  | D2 (Gy) | 68.60 (68.35-68.80) | 67.57 (65.09-68.67) | 66.57 (63.94-67.65) |
|  | V70 (%) | 0.06 (0.00-0.16) | 0.00 (0.00-0.00) | 0.00 (0.00-0.00) |
|  | V50 (%) | 23.98 (19.92-29.18) | 20.99 (15.40-24.01) | 19.09 (14.12-24.09) |
|  | V40 (%) | 31.24 (28.14-43.36) | 32.16 (24.65-40.01) | 30.50 (24.45-42.81) |
|  | EUD (Gy) | 50.92 (49.18-52.08) | 47.62 (46.25-50.45) | 46.46 (45.80-49.35) |
|  | gEUD (Gy) | 49.97 (48.05-50.92) | 45.41 (44.16-49.26) | 44.60 (42.57-47.50) |
|  |  |  |  |  |
| Bladder | D50 (Gy) | 20.77 (14.65-38.51) | 25.66 (18.16-50.91) | 31.62 (21.14-48.83) |
|  | Dmean (Gy) | 31.50 (26.43-40.37) | 35.27 (27.71-46.78) | 37.22 (28.61-46.16) |
|  | D2 (Gy) | 69.30 (68.98-69.98) | 69.41 (27.71-46.78) | 69.21 (68.07-69.79) |
|  | V70 (%) | 0.47 (0.13-1.83) | 0.53 (0.00-0.02) | 0.17 (0.00-0.47) |
|  | V55 (%) | 33.86 (23.12-41.95) | 35.19 (21.71-47.04) | 37.26 (21.91-45.09) |
|  | V45 (%) | 37.89 (27.40-46.73) | 39.98 (27.02-54.71) | 42.84 (28.05-53.70) |
|  | EUD (Gy) | 56.28 (52.64-58.52) | 57.04 (51.87-59.95) | 57.46 (51.57-59.20) |
|  | gEUD (Gy) | 55.99 (52.16-58.41) | 56.94 (51.34-59.51) | 56.96 (50.83-58.71) |

CTV – clinical target volume; EUD – equivalent uniform dose; gEUD – generalized equivalent uniform dose; CI – conformity index; COIN – conformal index
